# Undiagnosed HIV Infections May Drive HIV Transmission in the Era of “Treat All”: A Deep-Sampling Molecular Network Study in Northeast China during 2016 to 2019

**DOI:** 10.3390/v14091895

**Published:** 2022-08-27

**Authors:** Bin Zhao, Yu Qiu, Wei Song, Mingming Kang, Xue Dong, Xin Li, Lu Wang, Jianmin Liu, Haibo Ding, Zhenxing Chu, Lin Wang, Wen Tian, Hong Shang, Xiaoxu Han

**Affiliations:** 1 NHC Key Laboratory of AIDS Immunology (China Medical University), National Clinical Research Center for Laboratory Medicine, The First Hospital of China Medical University, Shenyang 110001, China; 2 Laboratory Medicine Innovation Unit, Chinese Academy of Medical Sciences, Shenyang 110001, China; 3 Key Laboratory of AIDS Immunology of Liaoning Province, Shenyang 110001, China; 4 Collaborative Innovation Center for Diagnosis and Treatment of Infectious Diseases, 79 Qingchun Street, Hangzhou 310003, China; 5 Department of Food Safety and Nutrition, Shenyang Center for Health Service and Administrative Law Enforcement (Shenyang Center for Disease Control and Prevention), Shenyang 110031, China

**Keywords:** HIV, treat all, molecular network, driving factor

## Abstract

Universal antiretroviral therapy (ART, “treat all”) was recommended by the World Health Organization in 2015; however, HIV-1 transmission is still ongoing. This study characterizes the drivers of HIV transmission in the “treat all” era. Demographic and clinical information and HIV *pol* gene were collected from all newly diagnosed cases in Shenyang, the largest city in Northeast China, during 2016 to 2019. Molecular networks were constructed based on genetic distance and logistic regression analysis was used to assess potential transmission source characteristics. The cumulative ART coverage in Shenyang increased significantly from 77.0% (485/630) in 2016 to 93.0% (2598/2794) in 2019 (*p* < 0.001). Molecular networks showed that recent HIV infections linked to untreated individuals decreased from 61.6% in 2017 to 28.9% in 2019, while linking to individuals with viral suppression (VS) increased from 9.0% to 49.0% during the same time frame (*p* < 0.001). Undiagnosed people living with HIV (PLWH) hidden behind the links between index cases and individuals with VS were likely to be male, younger than 25 years of age, with Manchu nationality (*p* < 0.05). HIV transmission has declined significantly in the era of “treat all”. Undiagnosed PLWH may drive HIV transmission and should be the target for early detection and intervention.

## 1. Introduction

In 2015, the World Health Organization (WHO) used high-quality evidence from randomized clinical trials and observational studies to recommend antiretroviral therapy (ART) for all people living with HIV (PLWH) regardless of clinical stage and CD4^+^ T cell count, marking the era of “treat all”. Although more than 130 countries had adopted the “treat all” policy [1], covering 27.5 million PLWH by 2020 [2], the UNAIDS goal of ending the AIDS epidemic by 2020 has not yet been reached [3]. In 2020, there were still 680,000 AIDS-related deaths and 1.5 million new infections worldwide [2]. Even in Western countries in Central Europe and North America, where ART coverage reached 83% in 2020, there were 67,000 new infections and 13,000 AIDS-related deaths [2]. In China, the “treat all” policy was implemented in 2016 [4], and by the end of 2019, 89.7% diagnosed PLWH had received ART, of whom 95.3% had durable viral suppression [5,6]. However, the number of newly reported HIV infections increased by nearly 50,000 compared with 2018 by the end of October 2019 [5,7]. Thus, the AIDS epidemic remains a major public health concern both in China and across the world. In the era of “treat all”, an understanding of the drivers of HIV transmission is urgently needed for the development of interventions to achieve the 2030 goal to end the epidemic.

Traditionally, the number of newly diagnosed HIV-infected individuals is usually used to reflect the epidemic; however, this indicator is not sensitive and accurate enough due to the detection scope and intensity. A laboratory-based detection method for recent HIV infections (RHI) has been implemented in various countries to estimate the HIV incidence [8]. A molecular network analysis method based on genetic distance was recently developed which is highly efficient at monitoring HIV transmission in large real-world cohorts [9]. Molecular network analysis can provide a better understanding on how HIV is spread between subpopulations [10] and define key populations that may be driving HIV transmission based on links within the network [11]. This analysis has also been used to assess the effectiveness of interventions. A recent molecular network study in China indicated that the “treat all” policy could reduce 53.6% second-generation transmission of HIV [12]. However, in the five years since this policy was implemented, no research in China has defined the current drivers of HIV transmission to guide targeted intervention to achieve the last mile of ending the HIV epidemic.

Shenyang is the largest city in northeastern China with a moderate HIV prevalence (>10,000 PLWH) [13], and MSM accounts for the largest proportion of HIV infections (81.7%) [14]. In this study, a whole population-based molecular network analysis was performed among all newly diagnosed individuals in Shenyang, from 2016, when the “treat all” policy was widely implemented, to 2019, in order to monitor the dynamics of the local HIV epidemic and determine the drivers of HIV transmission in the “treat all” era. The findings of this study can be further extended to areas with similar characteristics of the HIV epidemic, guiding targeted detection and interventions.

## 2. Materials and Methods

### 2.1. Study Design

A real-world observational cohort study was performed in Shenyang, the largest industrialized city in Northeast China, where about 1000 cases are newly diagnosed with HIV infection each year [15]. Individuals who met the following standards were enrolled in this study: (a) screened and diagnosed with HIV infection between 2016 and 2019, (b) ≥18 years of age, (c) self-reported HIV treatment-naïve before HIV diagnosis, and (d) having both *pol* gene sequence and demographic information records. If more than one sequence was available for an individual, only the earliest records were included in this analysis. The study was approved by the Institutional Review Board of China Medical University.

### 2.2. Data and Sample Collection

Demographic (age at diagnosis, gender, ethnicity, marital status, education), epidemiologic (transmission route, and date of diagnosis), and clinical (viral load [VL] and CD4 + T cell count) information along with cryopreserved plasma samples were collected at the time of HIV diagnosis by the Shenyang Center for Disease Control and Prevention (CDC) and Red Ribbon Outpatient of the First Affiliated Hospital of China Medical University. Follow-up data (date of ART initiation, survival status, VL, and CD4 + T cell counts determined at least once a year) was also collected for subsequent analysis.

### 2.3. Definition of ART Status

For all persons receiving ART, viral suppression (VS) was defined as the most recent VL ≤ 200 copies/mL, and unsuppressed viremia was defined as VL > 200 copies/mL. Virological failure was defined as VL > 200 copies/mL after over 48 weeks on ART. A blip was defined as VL > 200 copies/mL preceded and followed by <200 copies/mL without changes to the ART regimen. The threshold of 200 copies/mL was identified according to Chinese guidelines for diagnosis and treatment of HIV/AIDS (2018) [16].

### 2.4. HIV-1 Limiting Antigen (LAg) Avidity Enzyme Immunoassay

The LAg-Avidity EIA kit (Maxim Biomedical, Inc., Rockville, MD, USA) was used to screen for recent HIV infection (within 6 months) in all newly diagnosed HIV-infected persons. If the normalized optical density (ODn) value was ≤2.0, triplicate confirmatory testing was performed. If the confirmatory ODn value was ≤1.5, the case was determined as RHI; otherwise, the case was determined as chronic HIV infection (CHI) [17].

### 2.5. Sequence Analysis

A 1035-bp fragment of the HIV *pol* gene (HXB2: 2268–3302) was collected using routine HIV drug resistance genotypic testing [18]. Sequences were aligned using RECall, an online sequence analysis tool [19]. Subtypes were determined using phylogenetic analyses based on the approximate maximum likelihood (ML) tree. The ML tree was constructed with GTR + I + G nucleotide substitution using IQ-Tree v2.0.5 [20], and a bootstrap value >90 was the criterion to determine lineage [20].

### 2.6. Estimating Effective Reproductive Number (Re) for Dominant Subtypes

To describe the dynamics of HIV transmission, the Re was calculated for the three main subtypes (CRF01_AE, CRF07_BC, and subtype B) in Shenyang from 2016 to 2019. The Birth-Death Skyline Serial (BDSKY) model was used to calculate Re in BEAST v.2.6.3 according to the previously described method [21,22,23]. Based on local epidemic conditions, the following BDSKY model priors were set: Re (LogNorm(0;1)), the rate of becoming non-infectious (Norm(2;0.001)), Origin (Uniform(0;20)), and sampling rate (Beta(10;10)). The convergence of the estimates was considered satisfactory when the effective sample size (ESS) was >200. The BDSKY Tools package was used in R v.4.0.2 to plot the trend of Re [22].

### 2.7. Identification and Analysis of the Molecular Networks

The molecular networks for the main subtypes (CRF01_AE, CRF07_BC, and subtype B), were constructed based on pairwise GD [24]. The optimal GD threshold for each subtype was used to construct high-resolution molecular networks [25]. Networks were visualized using Cytoscape v3.8.2 [26].

Firstly, the sequences of cases newly diagnosed in 2016 were used to construct the baseline molecular networks, and the sequences of cases newly diagnosed in 2017 were added to the baseline networks. The RHI in 2017 were regarded as index cases [27], and individuals linked to the index cases were defined as potential transmission sources. Next, transmission direction between the index cases and potential transmission sources was determined according to the infection status and the date of HIV diagnosis. The direction of transmission could be determined if the potential transmission source was a CHI diagnosed before index cases or an RHI diagnosed ≥ 180 days earlier than the index case. In this case, the contribution of the potential transmission source to the transmission link was defined as 1. If the direction could not be determined, the contribution of the potential transmission source was defined as 1/2. Supplemental Figure S1 shows the analytic process as a flow diagram and supplemental Table S1 shows the results. ART status and the virological response of potential transmission sources were estimated using the most recent VL results before the index case diagnosis date [28], and the cases were divided into four groups: untreated (including previously diagnosed untreated cases, newly diagnosed untreated CHI and newly diagnosed untreated RHI), VS, unsuppressed, and unavailable VL. The sequences of newly diagnosed cases in 2018 and 2019 were successively added to the molecular network and analyzed in the same way.

### 2.8. Statistical Analysis

Continuous variables were represented by the median and interquartile range (IQR), and categorical variables were represented as numbers and percentages. The Chi-square test was used to compare the percentage and non-normal distribution data. Univariate and multivariate logistic regression analyses were performed to identify risk factors for HIV transmission, generating adjust odds ratios (AORs) and 95% confidence intervals (CIs). A *p*-value <0.05 was considered statistically significant, and a *p*-value < 0.1 was considered marginally statistically significant. All analyses were performed using SPSS software version 25.0 (SPSS Inc., Chicago, IL, USA).

## 3. Results

### 3.1. Demographic Characteristics and ART Status of the Cases

During 2016 to 2019, 2882 newly diagnosed cases (84.0%) were recruited from a total of 3434 in Shenyang. Most were male (93.2%, 2686/2882), and unmarried (62.7%, 1818/2882). The median age was 32 years (IQR = 26–44). Most cases (82.1%, 2367/2882) were MSM. One-third (33.1%, 2677/2882) were defined as RHI at the time of diagnosis (Table 1).

**Figure 1:**
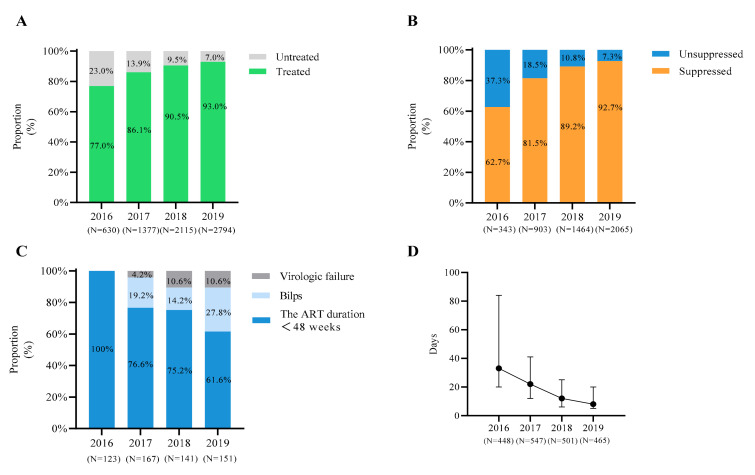
Status of ART among newly diagnosed PLWH in Shenyang during 2016 to 2019: (**A**) the green regions represent ART cumulative coverage among surviving HIV-infected individuals; (**B**) individuals receiving ART are grouped based on VL data, with each color representing a different group; (**C**) the histogram shows the details of the unsuppressed group; (**D**) the black dots represent the number of median days between diagnosis and ART initiation for newly diagnosed PLWH, and the short lines represent the first and third quartiles, respectively.

Since the “treat all” policy was implemented in Shenyang in 2016, the cumulative ART coverage of PLWH (the number of patients receiving ART/the number of patients with available ART information) increased significantly from 77.0% (485/630) in 2016, 86.1% (1186/1377) in 2017, 90.5% (1914/2115) in 2018 to 93.0% (2598/2794, *p* < 0.001) in 2019. For PLWH who received ART and had available VL, the cumulative proportion of VS also increased significantly from 62.7% (215/343) in 2016 to 92.7% (1914/2065, *p* < 0.001) in 2019. The median time from diagnosis to ART initiation was shortened from 33 days (IQR: 20–84 days, *N* = 448) in 2016 to 8 days (IQR: 5–20 days, *N* = 465) in 2019 (*p* < 0.001) (Figure 1). 

### 3.2. Re of Dominant HIV Strains

Given that CRF01_AE (70.0%, 2019/2882), CRF07_BC (18.3%, 526/2882), and subtype B (4.6%, 132/2882) accounted for 92.9% (2677/2882) of all cases in this study, we analyzed the Re of these subtypes to assess HIV epidemic. It was shown that the Re of all three strains declined significantly from two to one in 2016 and then fluctuated around one (Supplemental Figure S2).

### 3.3. Molecular Networks of Dominant HIV Strains

Molecular networks of the three main subtypes were constructed using 0.007 subs/site as the optimal GD threshold [25]. Overall, 853 (42.2%) CRF01_AE sequences formed 230 clusters (size range: 2 to 99), including 330 RHIs, 252 (47.9%) CRF07_BC sequences formed 55 clusters (size range: 2 to 70), including 96 RHIs, and 51 (38.6%) B sequences formed 18 clusters (size range: 2 to 8), including 15 RHIs. MSM was the dominant high-risk population among RHIs (85.0%, 375/441) (Supplemental Figure S3A–C).

The clustering rate of RHIs in the three subtypes was used to explore HIV transmission trends. The annual RHI clustering rate of CRF01_AE did not change significantly (42.5–52.9%); however, the RHI clustering rate of CRF07_BC dropped significantly from 55.6% to 29.5% (*p* = 0.005). For subtype B, the RHI clustering rate fluctuated greatly from 2016 to 2019 (62.5–0.0%) due to the small sample size of RHI (*N* = 31) and all the RHIs in 2019 (*N* = 3) were not included in the networks (Supplemental Figure S3D).

**Figure 2:**
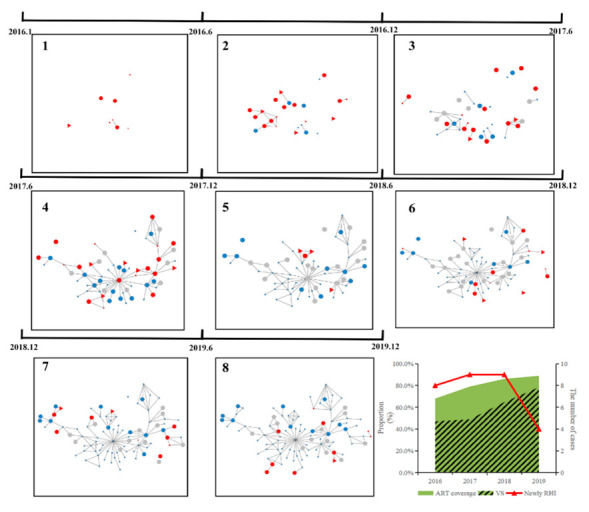
Dynamic analysis of the largest CRF01_AE cluster expansion and antiretroviral therapy during 2016 to 2019. The pictures 1–8 shows the outbreak process of the largest CRF01_AE cluster from Jan 2016 to Dec 2019. Red represents newly diagnosed PLWH, and blue represents those previously diagnosed. Circles represent CHI, and triangles represent RHI. Node size represents each case’s most recent VL. The gray circle represents individuals previously diagnosed with VL-unavailable. The combination diagram shows the specific data for this cluster. Green represents ART coverage, and blue represents the proportion of individuals with VS. The red line shows the change in the number of newly diagnosed RHI.

Expansion of the largest molecular cluster (*N* = 99) in the networks was used as a typical example to show the impact of the “treat all” policy on HIV transmission. Both the cumulative ART coverage of this cluster (from 68.0% in 2016 to 88.9% in 2019) and the cumulative proportion of VS (from 47.1% in 2016 to 78.4% in 2019) increased significantly (*p* < 0.05). The number of RHIs in this cluster was stable from 2016 to 2018 and decreased in 2019 (Figure 2).

### 3.4. Drivers of HIV Transmission

**Figure 3:**
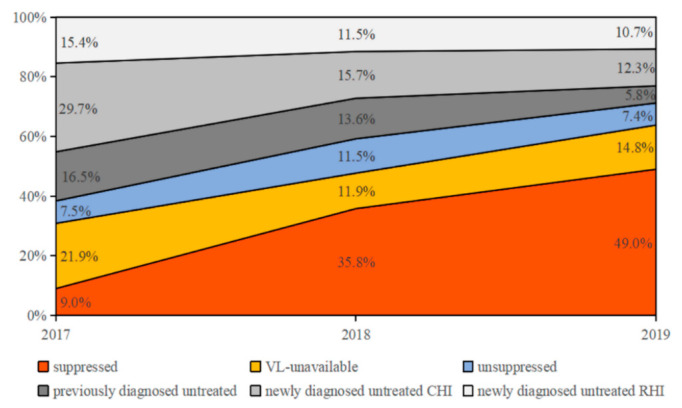
The contribution of different ART groups to RHI links. Each color represents the ART status of each potential transmission source.

To further explore the drivers of HIV transmission, we analyzed ART status and the virological response of potential transmission sources in the networks. The proportion of untreated persons linking to index cases dropped sharply from 61.6% (including previously diagnosed untreated cases [16.5%] and newly diagnosed untreated CHI [29.7%] and RHI [15.4%]) in 2017 to 28.8% (including previously diagnosed untreated cases [5.8%] and newly diagnosed untreated CHI [12.3%] and RHI [10.7%]) in 2019 (*p* < 0.001), in which the links between index cases and newly diagnosed untreated CHI accounting for the highest proportion, 48.2% (29.7%/61.6%) in 2017 and 42.7% (12.3%/28.8%) in 2019 (Figure 3). During the same period, the proportion of links between the index cases and VS persons increased rapidly from 9.0% in 2017 to 49.0% in 2019 (*p* < 0.001). Although the proportion of VL-unavailable persons linking to index cases decreased (from 21.9% to 14.8%, *p* = 0.044), the proportion of unsuppressed persons did not change significantly (from 7.5% to 7.4%).

The most likely explanation for the above results is that the undiagnosed PLWH hidden behind the links between VS and index cases may be the source for transmission of HIV to index cases. This is further supported by the high percentage of newly diagnosed untreated CHI that is linked to index cases (from 29.7% in 2017 to 12.3% in 2019), as the undiagnosed PLWH could have transmitted HIV to index cases prior to diagnosis. The demographic characteristics of individuals with VS and newly diagnosed untreated CHI (*N* = 204) were obtained through comparison with CHI outside molecular networks (*N* = 1105). These characteristics included male (AOR = 3.332, 95%CI = 1.205–9.211, *p* = 0.020), <25 years of age (AOR = 1.596, 95%CI = 1.090–2.336, *p* = 0.016), Manchu nationality (AOR = 1.746, 95%CI = 1.121–2.719, *p* = 0.014), have a history of injection drug use (IDU) (AOR = 3.765, 95%CI = 1.201–11.801, *p* = 0.023) (Table 2).

## 4. Discussion

This study supported that the “treat all” policy significantly prevents HIV transmission through a real-world observation of deeply sampled population-level data. More importantly, findings reveal that RHIs are increasingly linked to individuals with VS in molecular networks, suggesting that undiagnosed PLWH is the main driving force of HIV transmission in the era of “treat all”. Therefore, on the basis of effective implementation of the “treat all” policy, priority intervention should focus on identifying undiagnosed HIV-infected persons and initiating ART for them as soon as possible.

Incidence is usually used to describe the occurrence of HIV infection, which may not be sensitive enough to reflect changes in the HIV epidemic because new diagnoses may not represent new infections due to the late diagnosis in many countries including China [29]. In this study, the number of newly diagnosed HIV infections did not change significantly each year, and only 33.1% of newly diagnosed infections were identified as RHI at diagnosis. Due to the genetic diversity of HIV-1, an accurate pattern of HIV-1 evolution and transmission could be obtained from sequences collected within a certain period [30]. The Re based on phylodynamics is shown to be reliable in many studies [22,31] and has been used to evaluate the effectiveness of interventions [22]. With a deep sampling of HIV-infected individuals in the local area, the Re of the main epidemic subtypes in Shenyang during 2016 to 2019 were shown to decline significantly, providing molecular evidence to support the effectiveness of the “treat all” policy, launched in 2016, in controlling HIV transmission.

The most important discovery of this study is that index cases were increasingly linked to PLWH with VS in the molecular networks. Prior research has indicated that undetectable equals untransmissible [32]. Moreover, in the molecular networks, two individuals may be linked by direct or indirect transmission relationships. So, the links between index cases and VS suggest that there may be undiagnosed PLWHs transmitting HIV to index cases in the same social contact networks, and this trend increases as HIV ART coverage and VS rates rise. The population of undiagnosed PLWH in China is still very large, with an estimated 360,000 undiagnosed PLWH in 2018 [29]. According to WHO data, 16% of PLWH in the world were still unaware of their infection status in 2020 [33]. In this study, links to newly diagnosed untreated CHI accounted for the highest proportion among links to untreated individuals, suggesting that HIV infection linked to index cases may have occurred prior to diagnosis. A recent modeling study illustrated that undiagnosed PLWH could cause more new HIV infections than untreated PLWH [34]. Results from this study combined with prior findings support that undiagnosed PLWH may drive continuous HIV transmission. A recent study of a Swiss HIV cohort reached similar conclusions [35], and our study further confirms the reliability of this hypothesis using real-world data and improved methodology. First, molecular networks were inferred using in-depth sampling of viral sequences (sampling depth = 84%), and second, molecular network analysis based on GD with relatively short time spans can reveal the recent virus transmission track [36]. Lastly, RHI determined using the HIV-1 LAg Avidity Enzyme Immunoassay improved the accuracy of HIV transmission analyses.

Although the scale-up of HIV testing in part drove the rise in newly diagnosed PLWH [29], HIV testing on populations with a higher risk of HIV infection may be better at finding undiagnosed PLWH. In this study, index cases were increasingly linked to the VS group, sharing similar demographic and social behavior characteristics with undiagnosed PLWH. Moreover, since the infection event of newly diagnosed CHI could occur during the undiagnosed period, they are also considered to have similar characteristics to undiagnosed PLWH. These two groups were more likely to be male, young (<25 years old), of Manchu nationality, and with a history of injection drug use. However, IDU may not be a very reliable risk factor because of the small number of IDU cases in this study (*n* = 44). Liaoning province is the main dwelling place of people of Manchu nationality [37]. Of the 44 IDUs in this study, the clustering rate reached 63.6%, indicating that HIV is closely related among IDUs and that IDU should be a focus for intervention at any time. In addition, young men are sexually active and more likely to have high-risk behaviors. A multicenter cross-sectional survey in China showed that young MSM (age < 25 years) had a significantly higher prevalence of HIV [38]. The strategies of HIV self-testing and pre-exposure prophylaxis (PrEP) are effective at increasing HIV diagnosis [39,40] and should be actively promoted among young MSM. To a lesser extent, untreated and viral unsuppressed PLWH also contributed to HIV transmission and should also be of concern.

Finally, with the increase of ART coverage, the emergence of HIV drug resistance is inevitable, which is the main threat to the successful adoption of ART. According to the sequence obtained in this study, the overall prevalence of transmitted drug resistance (TDR) of Shenyang has reached 9.1% (moderately prevalent) [14]. Molecular network analysis revealed that TDR strains had been transmitted among MSM in Shenyang [14]. These results suggested that it is necessary to carry out baseline HIV drug resistance testing to monitor the transmission of HIV drug-resistant strains in real time while expanding the scope of ART.

There were still some limitations to this study. First, HIV transmission may be underestimated because the molecular networks of only three major epidemic strains were analyzed. Second, VL data of some individuals were unavailable, which may lead to underestimates of the VS rate and effectiveness of ART. Finally, the lack of high-risk behavior data for PLWH, such as the number of sexual partners and prevalence of syphilis co-infection, made it difficult to fully analyze the risk of HIV transmission within the molecular network.

## 5. Conclusions

The HIV epidemic has significantly declined since implementation of the “treat all” policy in Northeast China, but HIV transmission has not been eradicated, and undiagnosed HIV-infected individuals hidden in the molecular network could drive HIV transmission in the era of “treat all”.

## Figures and Tables

**Table 1:** Characteristics of individuals in this study.

Characteristics	Total (*N*, %)	2016 (*N*, %)	2017 (*N*, %)	2018 (*N*, %)	2019 *N* (%)
Total	2882	666(23.1%)	755(26.2%)	750(26.0%)	711(25.7%)
**Age**					
Median (IQR)	32(26–44)	30(25–44)	33(26–46)	31(26–43)	32(26–45)
≤25	710(24.6)	183(27.5)	178(23.6)	184(24.5)	165(23.2)
>25	2172(75.4)	483(72.5)	577(76.4)	566(75.5)	546(76.8)
**Gender**					
Male	2686(93.2)	633(95.0)	700(92.7)	706(94.1)	647(91.0)
Female	196(6.8)	33(5.0)	55(7.3)	44(5.9)	64(9.0)
**Ethnicity**					
Han	2482(86.1)	579(86.9)	660(87.4)	642(85.6)	601(84.5)
Others	400(13.9)	87(13.1)	95(12.6)	108(14.4)	110(15.5)
**Marital status**					
Unmarried	1818(62.7)	428(64.3)	459(60.8)	479(63.9)	442(62.2)
Married	548(19.0)	102(15.3)	161(21.3)	132(17.6)	153(21.5)
Divorced	519(18.0)	136(20.4)	134(17.8)	133(17.7)	116(16.3)
Not available	7(0.3)	0(0.0)	1(0.1)	6(0.8)	0(0.0)
**Education**					
Senior high school and above	2019(70.0)	483(72.5)	532(70.5)	522(69.6)	482(67.8)
Junior high school and below	853(29.6)	181(27.2)	221(29.2)	222(29.6)	229(32.2)
Not available	10(0.4)	2(0.3)	2(0.3)	6(0.8)	0(0.0)
**Infection Route**					
Men who have sex with men	2367(82.1)	592(88.9)	616(81.6)	598(79.8)	561(78.9)
Heterosexual transmission	434(15.1)	63(9.5)	115(15.2)	118(15.7)	138(19.4)
Injection drug users	44(1.5)	9(1.3)	13(1.7)	13(1.7)	9(1.3)
Other/Not available	37(1.3)	2(0.3)	11(1.5)	21(2.8)	3(0.4)
**Subtype**					
CRF01_AE	2019(70.0)	471(70.7)	554(73.4)	517(68.9)	477(67.1)
CRF07_BC	526(18.3)	120(18.0)	125(16.5)	148(19.8)	133(18.7)
B	132(4.6)	33(5.0)	31(4.1)	31(4.1)	37(5.2)
Others	205(7.1)	42(6.3)	45(6.0)	54(7.2)	64(9.0)
**Infection status**					
Recent HIV infection	953(33.1)	205(30.8)	252(33.4)	295(39.3)	201(28.3)
Chronic HIV infection	1868(64.8)	435(65.3)	494(65.4)	450(60.0)	489(68.7)
Not available	61(2.1)	26(3.9)	9(1.2)	5(0.7)	21(3.0)
**CD4+T cell (cells/μL)**					
<350	1244(43.2)	288(43.2)	340(45.0)	410(54.6)	206(29.0)
350–500	419(14.5)	90(13.5)	122(16.2)	131(17.5)	76(10.7)
>500	323(11.2)	81(12.3)	73(9.7)	108(14.4)	61(8.6)
Not available	896(31.1)	207(31.0)	220(29.1)	101(13.5)	368(51.7)
**Viral Load (copies/mL)**					
<10,000	194(6.7)	51(7.7)	60(7.9)	48(6.4)	35(4.9)
≥10,000	1383(48.0)	357(53.6)	339(44.9)	391(52.1)	296(41.7)
Not available	1305(45.3)	258(38.7)	356(47.2)	311(41.5)	380(53.4)

**Table 2:** Characteristics of undiagnosed HIV-infected individuals.

Characteristics	*N*	(%)	Univariate Analyses			Multivariate Analyses		
			Odds Ratio	95% Confidence Interval	*p-*Value	Adjusted Odds Ratio	95% Confidence Interval	*p*-Value
**Gender**								
Female	86	6.6	Ref			Ref		
Male	1223	93.4	3.148	1.26–7.867	0.014	3.332	1.205–9.211	0.020
**Age at diagnosis**								
≥25	1052	80.4	Ref			Ref		
<25	255	19.5	1.667	1.18–2.353	0.004	1.596	1.090–2.336	0.016
**Marriage**								
Unmarried	806	61.6	Ref			Ref		
Married	268	20.5	0.668	0.442–1.009	0.055	0.867	0.552–1.362	0.537
Divorced/Widowed	231	17.7	0.91	0.609–1.357	0.642	1.115	0.720–1.728	0.625
**Nationality**								
Han	1134	86.6	Ref			Ref		
Manchu	128	9.8	1.851	1.196–2.863	0.006	1.746	1.121–2.719	0.014
Others	46	3.5	0.869	0.363–2.081	0.752	0.837	0.348–2.018	0.692
**Infection Route**								
Men who have sex with men	1084	82.8	Ref			Ref		
Heterosexual transmission	210	16.1	0.707	0.452–1.106	0.129	1.064	0.642–1.764	0.808
Injection drug users	15	1.1	2.615	0.883–7.744	0.083	3.765	1.201–11.801	0.023

## Data Availability

Publicly available datasets were analyzed in this study. This data can be found here: https://doi.org/10.3389/fmicb.2021.797771 (accessed on 7 January 2022).

## Supplementary Materials

The following supporting information can be downloaded at: https://www.mdpi.com/article/10.3390/v14091895/s1, Figure S1: Flow diagram depicts the process of determining HIV transmission direction; Figure S2: Re trends for the three dominant subtypes in Shenyang; Figure S3: Overview of molecular networks for CRF01_AE, CRF07_BC and subtype B, and the cluster rate of recent HIV infection in the three main subtypes in Shenyang during 2016 to 2019.
