# Supplementary material for: Undiagnosed HIV Infections May Drive HIV Transmission in the Era of “Treat All”: A Deep-Sampling Molecular Network Study in Northeast China during 2016 to 2019
Source: Viruses. 2022 Aug 27;14(9):1895. doi: 10.3390/v14091895 (PMC9502473; doi:10.3390/v14091895)
Supplement: Supplementary file 1 [file viruses-14-01895-s001.zip › viruses-1840531-supplementary.pdf]

viruses

MDPI

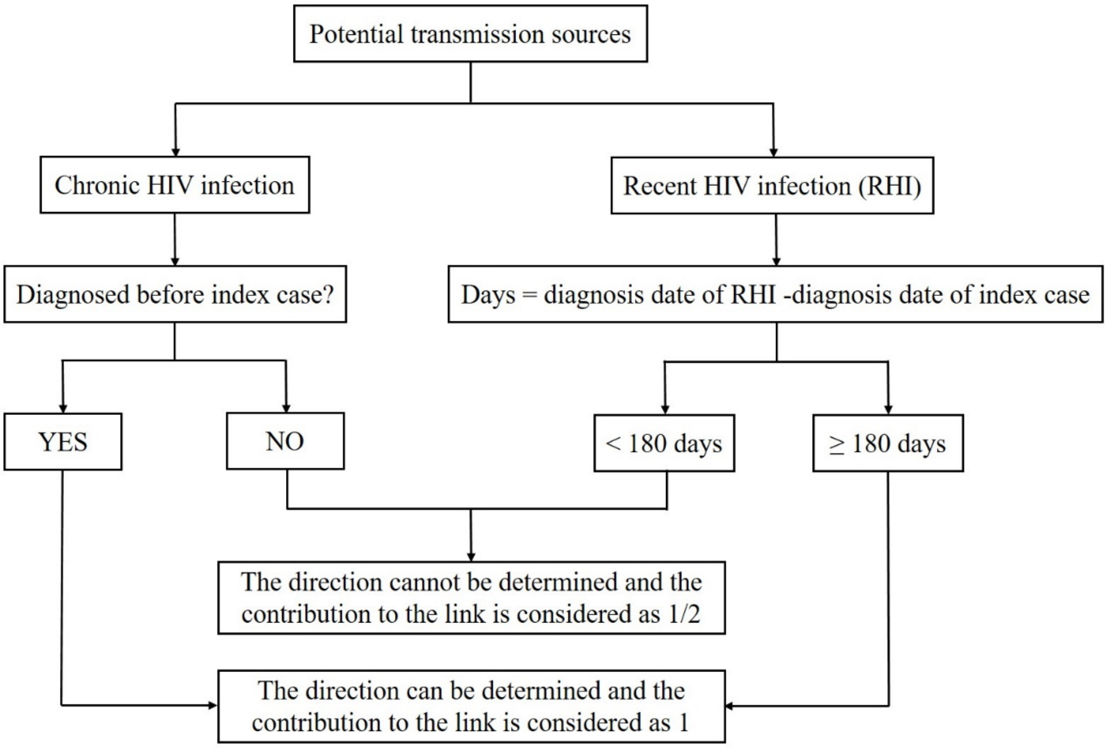

Figure S1. Flow diagram depicts the process of determining HIV transmission direction.

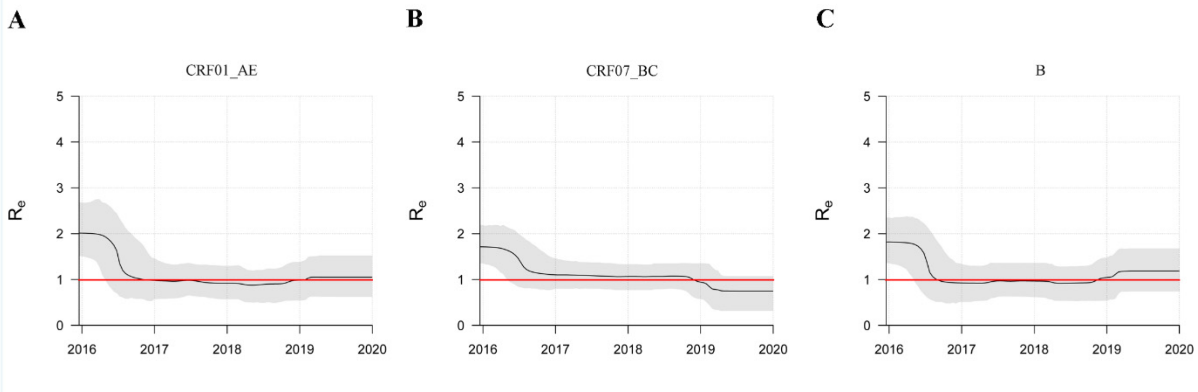

Figure S2. Re trends for the three dominant subtypes in Shenyang.

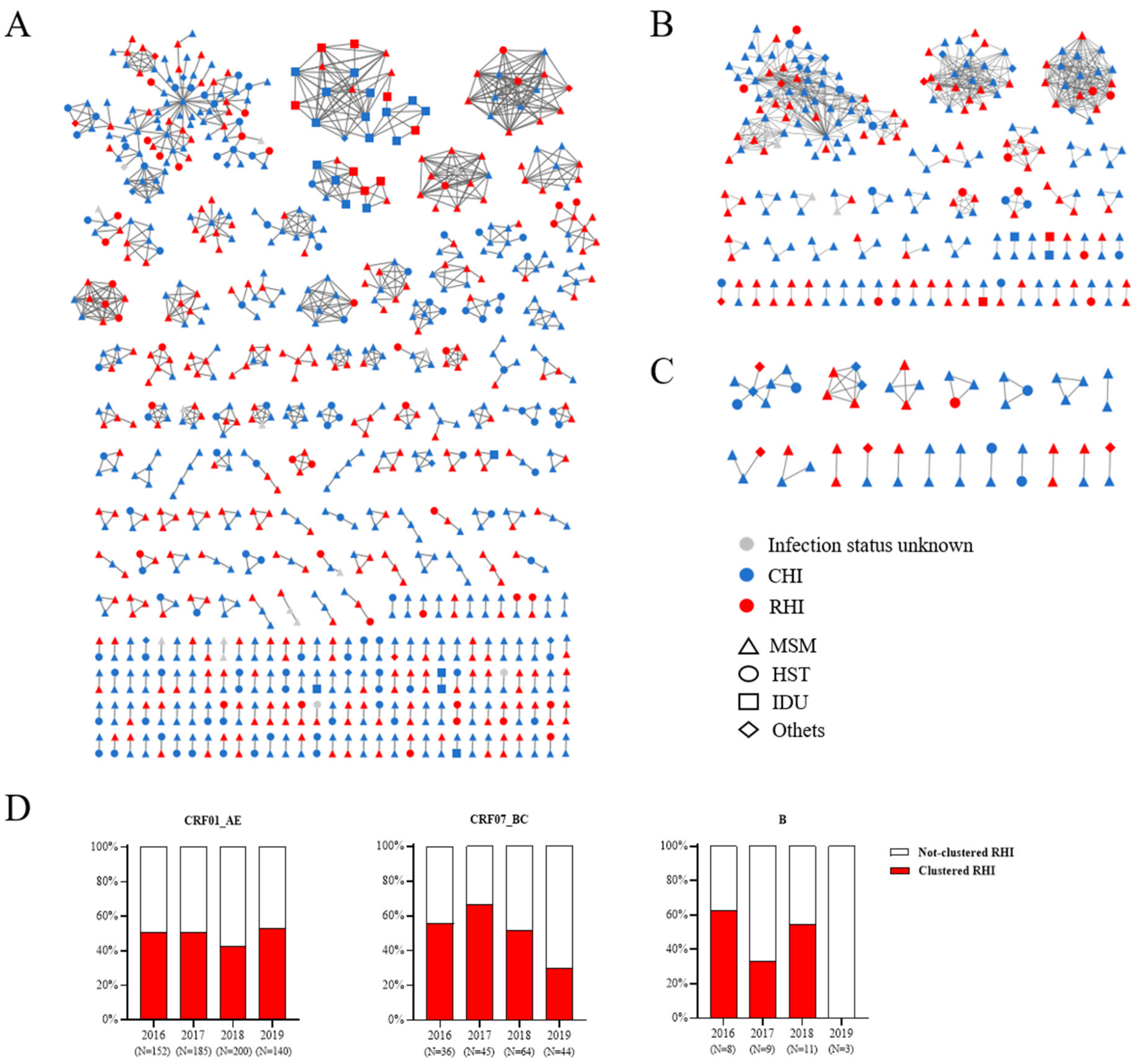

Figure S3. Overview of molecular networks for CRF01_AE (A), CRF07_BC (B) and subtype B (C), and the cluster rate of recent HIV infection in the three main subtypes (D) in Shenyang during 2016 to 2019.CHI:chronic HIV infection, RHI: recent HIV infection, MSM: men who have sex with men, HST: heterosexual transmission, IDU: injection drug user.
